# Metastatic-initiating cells and lipid metabolism

**DOI:** 10.15698/cst2017.12.113

**Published:** 2017-11-21

**Authors:** Salvador Aznar Benitah

**Affiliations:** 1 Institute for Research in Biomedicine (IRB Barcelona), The Barcelona Institute of Science and Technology (BIST), Barcelona, Spain.; 2 Catalan Institution for Research and Advanced Studies (ICREA), Barcelona, Spain.

**Keywords:** metastasis, metastatic-initiating cells, lipid metabolism, CD36, metabolic rewiring, diet, anti-metastatic therapy

## Abstract

The identity of the cells responsible for initiating and promoting metastasis has been historically elusive. Consequently, this has hampered our ability to develop specific anti-metastatic treatments, resulting in the majority of metastatic cancers remaining clinically untreatable. Furthermore, advances in genome sequencing indicate that the acquisition of metastatic competency does not seem to involve the accumulation of *de novo* mutations, making it difficult to understand why some tumours become metastatic while others do not. We have recently identified metastatic-initiating cells, and described how they specifically rely on fatty acid uptake and lipid metabolism to promote metastasis. This intriguing finding indicates that external influences, such as those derived from our diet, exert a strong influence on tumour progression, and that such dietary factors could be therapeutically modulated if understood. In this *News and Thoughts,* I will comment on recent findings regarding how and why lipid metabolism modulates the behaviour of metastatic cells, and how this knowledge can be harnessed to devise new and specific anti-metastatic therapies.

## INTRODUCTION

Metastasis is an inefficient process at the cellular level, yet it is the leading cause of cancer-related deaths 1. Despite intense investigation, the identity of the tumor cells with the capacity to colonize distant sites has remained historically elusive, hampering our ability to develop effective anti-metastatic therapies. Recent studies based on *in vivo* lineage-tracing have shown that cells that initiate and maintain primary tumors arise primarily from adult stem cells that have accumulated genetic and epigenetic alterations 2,3,4,5,6,7. However, are these tumor-initiating cells also responsible for generating metastases? That is, are tumor-initiating cells and metastasis-initiating cells two sides of the same coin, or are they two independent populations? Furthermore, why some tumor cells acquire metastatic competency while others remain within the primary tumor lesion?

The field of cancer research has historically posited that tumor progression entails the progressive accumulation of mutations, and that acquiring the ability to egress the primary tumor and colonize distant sites occurs over long periods of time. However, recent evidence suggests otherwise. For instance, careful *in vivo* studies suggest that disseminated cells can reach distant sites very early during primary tumor growth, yet can remain dormant—and at the moment, untreatable—for long periods of up to years before generating metastases that often become fatal 8,9. Furthermore, whole genome sequencing of an increasing number of human tumor samples indicates that primary tumors and metastatic lesions harbor more-or-less the same set of driver mutations 10,11,12. These results should not be misinterpreted, as they do not mean that there are no specific driver mutations associated to metastatic competency. What they suggest is that the process of metastasis not only relies on the early accumulation of the panoply of driver mutations, but also on a combined set of signaling and epigenetic pro-metastatic elements. Critically, this also suggests that factors derived from our lifestyle might exert a strong influence on tumor progression, and that such factors could be therapeutically targeted if understood. Yet, we still know very little about the nature of the signals that promote metastasis, their origin, how they promote colonization of distant sites, or why not all tumor cells respond to them similarly.

## IDENTIFICATION OF METASTATIC-INITIATING CELLS AND THEIR LINK TO LIPID METABOLISM

**Figure 1:**
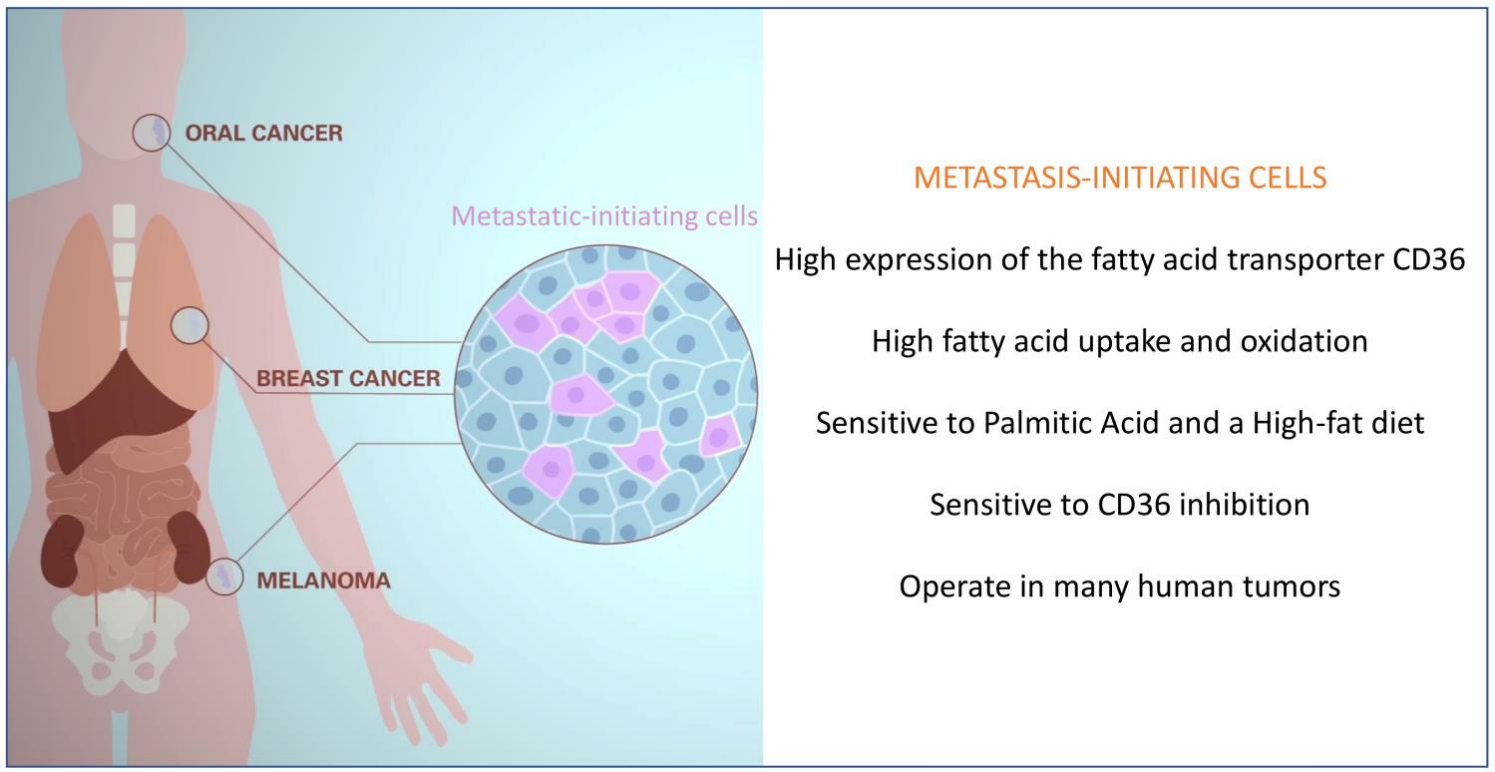
FIGURE 1: Diagram summarizing key aspects of metastatic-initiating cells identified in a variety of human tumours. Metastatic-initiating cells are characterized by their high expression of the fatty acid transporter CD36 and a prominent lipid metabolism. This makes metastatic cells highly responsive and sensitive to dietary fatty acids, such as palmitic acid, and dependent on the activity of CD36 to metastasize to different organs (*i.e.* lymph nodes, lungs, liver, bone).

As a first step towards addressing these critical questions, we have recently identified a population of metastasis-initiating cells in several types of human tumors, and have determined that these are highly sensitive to dietary fatty acids 13 (**Figure 1**). This has allowed us to start identifying the intriguing characteristics that distinguish metastatic from non-metastatic cells. Specifically, we have found that**: i)** metastasis-initiating cells remain relatively quiescent in the primary tumor, but become highly proliferative during the initial stages of metastatic colonization; **ii)** upon orthotopic transplantation, metastasis-initiating cells initiate primary lesions as efficiently as their previously identified primary tumor-initiating counterparts, yet differ in that they are exclusive in their ability to generate metastasis; **iii)** metastatic-initiating cells express high levels of the fatty acid translocase CD36, and are characterized by a distinct lipid metabolic signature related to lipid uptake, fatty acid beta-oxidation (*i.e.* withdrawing energy from lipids), lipid biosynthesis, and lipid storage; **iv)** the metastatic potential of metastatic-initiating cells is boosted by a high-fat diet or exposure to high concentrations of palmitic acid. Consequently, the predisposition of tumors to develop metastasis might be directly linked to the content of fat in our diet; **v)** importantly, metastatic-initiating cells are therapeutically sensitive to CD36 inhibition, which almost completely abolishes their metastatic potential in orthotopic models of melanoma, and oral, breast, and ovarian cancers (so far tested) without significantly affecting their ability to generate primary tumors, and without overt systemic side effects; and **vi)** high expression of CD36+ correlates with poor prognosis in melanoma, and ovarian, hepatocellular, breast, head and neck, lung, pancreatic and bladder cancers, suggesting that these cells operate in many different types of tumors 13,14 (**Figure 1**). Of note, although the signature of CD36+ cells does not seem to be strongly associated with the expression of epithelial to mesenchymal transition (EMT) genes, fatty acid uptake by CD36 and the fatty acid binding proteins FABP1 and FABP4 induce EMT in liver cancer cells, thereby increasing their invasive capacity 13,15.

## SOURCES AND MECHANISMS OF LIPID METABOLIC REWIRING IN METASTATIC CELLS

The results described above indicate that metastatic cells are very sensitive to lipid metabolism, unearthing a previously overlooked link between how much fat, and the type of fat, we eat and metastasis. However, many aspects of this metabolic switch remain unknown. First, why do metastatic-initiating cells respond to certain fatty acids such as palmitic acid, and how does this metabolism mechanistically affect their ability to colonize distant organs? Although the precise answer is unknown, it is likely that cancer cells require large amounts of energy to withstand the selective pressure against colonizing distant sites. For instance, highly aggressive triple negative breast cancer cells boost mitochondrial fatty acid beta-oxidation to obtain elevated amounts of acetyl-CoA that feed into the TCA cycle, thereby generating large quantities of ATP 16. Interestingly, this metabolic switch is induced by Myc and Src, two well-known regulators of cancer progression and metastasis 16,17,18. Besides Myc and Src, other signalling pathways might operate to allow cancer cells to elevate lipid metabolism. For instance, our recent findings indicate that epidermal stem cells lacking the *de novo* DNA methyltransferase Dnmt3a upregulate the expression of the lipid signature associated with CD36+ metastatic-initiating cells, and are consequently much more predisposed to generate metastatic squamous tumours 19,20. Considering that Dnmt3a is one of the most mutated genes in human cancers, it will be interesting to study in the future the interplay between dietary lipids and loss-of-function mutations in this epigenetic regulator towards metastatic progression 11,21.

Besides our little knowledge of the cell intrinsic energetic and signalling changes lipid metabolism triggers in cancer cells to facilitate metastasis, our understanding of the factors that drive this metabolic switch is also still quite limited. One possible source of lipid metabolism rewiring could come from the fact that metastatic cells might adjust their metabolic state (*i.e.* metabolic plasticity) to the systemic and local changes they encounter as they egress the primary lesion and reach a distant site. For instance, as tumours grow, their capillary network becomes unstructured and generates areas of low oxygen, a phenomenon known as hypoxia. In addition, as metastatic cells arrive to distant organs, they initially lack a proper vasculature ensuring the oxygen supply necessary for tumour re-growth. Interestingly, in certain tumours, hypoxia can promote using alternative carbon sources to glucose, such as acetate, to sustain fatty acid synthesis. This lipid metabolic switch is associated to activation of the synthase ACSS2, whose expression is specifically increased in metastatic cells 22. In addition, hypoxia drives mammary gland tumours to accumulate lipids in the form of lipid droplets through the uptake of fatty acids via the FABP7 and FABP3 lipid transporters. These lipid reservoirs can in turn sustain cancer survival by providing ATP production and building blocks for cell division via fatty acid beta-oxidation in the mitochondria 23.

Besides hypoxia-driven lipid metabolism, metastatic cells might obtain fat as an energy source by hijacking it from the cells they interact with during the metastatic journey. Two interesting examples support the notion of lipid metabolic coupling between cancer cells and their local cellular environment. For instance, metastatic ovarian cancer cells absorb lipids from the ommentum fat layer, one of the first sites they colonize, thereby ensuring a plentiful supply of energy required for surviving the colonization process 24,25. Interestingly, metastatic ovarian cancer cells also express high levels of the enzyme monoacylglycerol lipase (MAGL), which releases free fatty acids from lipids 26. Blockade of MAGL impairs tumour growth and migration, which are rescued by free fatty acids present in a high-fat diet, further underscoring the importance of dietary lipids in promoting malignancy of cancer cells 26. A second example of tumour-stroma metabolic coupling is found in leukaemia. Although leukaemia cells do not metastasize, a recent elegant report has shown that chronic myeloid leukaemia (CML) stem cells specifically associate with the gonadal adipose tissue when challenged with chemotherapy. Similar to the traits we have identified in CD36+ metastatic-initiating cells of solid tumors 13, chemotherapy-resistant leukaemia stem cells remain quiescent when bound to the adipose tissue, secrete pro-inflammatory cytokines such as TNF-alpha and IL-1alpha to stimulate lipolysis in gonadal adipocytes, and up-take and oxidize the released free fatty acids through CD36 27. This metabolic coupling in turn confers leukaemia stem cells with the energetic supply and anti-oxidative potential to resist chemotherapy.

## CONCLUDING REMARKS: THERAPEUTIC OPPORTUNITIES REGARDING LIPID METABOLISM IN METASTASIS

In conclusion, the ability of metastatic cells to engage in a high lipid metabolic state when confronted with different conditions and environments, strongly contributes to tumour progression. We find these findings very interesting scientifically, albeit socially worrying considering the high amount of added fat that is consumed regularly through processed food in established and newly industrialized countries. The percentage of clinically obese and overweight people has nearly doubled in the last 30 years, with more than 600 million adults considered obese, and 41 million children under the age of 5 overweight or obese, in 2014 (http://www.who.int/mediacentre/factsheets/fs311 . The phenomenon is occurring worldwide, and even in countries that have historically favored a Mediterranean diet, underscoring the fact that eating habits have dramatically changed with industrialization.

On the other hand, and notwithstanding these worrying figures, the connection between lipid metabolism and metastasis might in fact offer new therapeutic avenues to prevent and treat metastatic cancers. First, public awareness can and should be increased to highlight the importance of drastically reducing the amount of added fat in the food we consume on a daily basis. As an example of the benefits of weight loss, a recent study shows that whereas obesity boosts lung metastasis of breast cancer cells by recruiting neutrophils to the pre-metastatic niche in the lungs, a loss of weight is sufficient to reverse this effect 28,29. Secondly, custom-made diets with low amounts of pro-metastatic lipids, such as palmitic acid, might be tested in the clinic as an additional measure to improve the outcome of patients with metastatic cancer. Furthermore, therapies targeted to different aspects of lipid metabolism are already being developed at the preclinical level with promising results, warranting some of them being tested in the clinic. For instance, targeting leukotriene production with a specific inhibitor strongly reduces metastatic spreading of breast cancer cells to the lungs, by preventing the recruitment of neutrophils that promote the formation of pro-metastatic niches 29. In addition, neutralizing antibodies against the fatty acid receptor CD36 almost completely prevent metastatic initiation, and significantly induce metastatic regression to different organs including lymph-nodes, lungs, liver, and bones, in models of melanoma, and oral and breast carcinomas 13. Importantly, systemic delivery of CD36 neutralizing antibodies is equally efficient in preventing metastasis in immunocompromised and immunocompetent mice, and does not result in any measurable side effect within the window of time required to obtain a potent anti-metastatic therapeutic activity. Other inhibitors such as those targeting the Acetyl-CoA Synthase (ACS), which is involved in fatty acid oxidation and is highly expressed in aggressive tumours, or the activity of Monoacylglicerol lipase (MAGL), which releases fatty acids from lipid reservoirs and is associated with breast cancer aggressiveness, have not been directly tested for their anti-metastatic effect, but show very promising anti-tumour activities 26. Future mechanistic studies, coupled to robust *in vivo* pre-clinical assays of metastasis, and clinical trials, will undoubtedly provide a deeper understanding of the relevance, and clinical potential, of lipid metabolism regarding the idiosyncrasies of metastatic-initiating cells.
